# Atypical Polypoid Adenomyofibroma of the Endometrium Presenting With Severe Abnormal Uterine Bleeding: A Case Report

**DOI:** 10.7759/cureus.114441

**Published:** 2026-08-12

**Authors:** Akash Patel, Yiru Wang, Mahsa Chitsaz

**Affiliations:** 1 Pathology, University of Illinois Chicago, Chicago, USA

**Keywords:** abnormal uterine bleeding, adenomyosis, atypical polypoid adenomyofibroma, biphasic uterine lesion, leiomyoma

## Abstract

Atypical polypoid adenomyoma (APA) of the endometrium is an exceedingly rare biphasic uterine lesion characterized by atypical endometrial glands embedded within fibromuscular stroma. The overlapping histologic features of premalignant and malignant endometrial lesions require accurate diagnosis to avoid overtreatment. APA is a polypoid lesion that consists of atypically complex endometrial glands that may or may not have squamous morules in a benign myomatous or fibromyomatous stroma. We present a case of a 42-year-old multiparous female with severe abnormal uterine bleeding resulting in symptomatic anemia requiring transfusion. Imaging suggested adenomyosis, and hysterectomy was performed in 2025 for definitive management. Histological examination revealed an APA arising in a secretory endometrium with coexisting adenomyosis and leiomyomata. In addition, a high-grade squamous intraepithelial lesion involving the cervix was identified. Postoperatively, the patient was placed on long-term surveillance with annual vaginal cytology for 25 years due to the presence of cervical HSIL. This case highlights the rarity of APA and underscores the importance of histologic evaluation when persistent abnormal uterine bleeding remains unexplained by imaging or biopsy.

## Introduction

Abnormal uterine bleeding (AUB) is a common gynecologic complaint among reproductive-aged females and may arise from structural, hormonal, or neoplastic etiologies [1]. Structural causes such as leiomyomas and adenomyosis account for many cases; however, rare endometrial lesions can occasionally contribute to severe bleeding and diagnostic uncertainty [1-3]. Among these uncommon entities are biphasic endometrial tumors composed of epithelial and stromal components, including atypical polypoid adenomyoma (APA) and related fibromuscular variants [4,5]. Atypical polypoid adenomyoma, also known as atypical polypoid adenomyofibroma, is a rare uterine tumor characterized by atypical endometrial glands embedded in a smooth muscle stroma, often accompanied by squamous morular metaplasia [3]. A systematic review of the literature identified 237 reported cases of APA across 11 retrospective studies, underscoring the rarity of this entity [6]. However, variants with prominent fibrous stromal components, such as APA, appear to be even rarer, with only isolated case reports and small case series reported in the literature [3,5,7]. Because of their scarcity and overlapping morphologic features with premalignant or malignant endometrial lesions, including endometrial intraepithelial neoplasia, adenosarcoma, and endometrioid carcinoma, accurate histopathologic identification is essential.

Clinically, these lesions most commonly present with AUB and may be mistaken for more common structural uterine pathologies such as endometrial polyps or adenomyosis on imaging studies. As a result, the diagnosis is frequently established only after hysteroscopic resection or hysterectomy when adequate tissue is available for histologic evaluation. The rarity of adenomyoma variants and their potential to mimic malignant disease make recognition of these lesions important for appropriate clinical management. Here, we present a case of APA identified in a hysterectomy specimen from a 42-year-old female with severe AUB requiring transfusion that was initially attributed to adenomyosis.

## Case presentation

A 42-year-old female with hypertension and active tobacco use presented for evaluation of persistent severe AUB. Her medical history was notable for a follicular variant of papillary thyroid carcinoma that was treated with thyroidectomy in 2013 and an adrenal cortical adenoma for which she underwent a unilateral adrenalectomy in 2024. She had experienced non-ST-elevation myocardial infarctions in 2024 and 2025. Coronary angiography performed at an outside hospital demonstrated non-obstructive coronary artery disease, and she was treated with amlodipine for presumed vasospastic angina.

**Figure 1:**
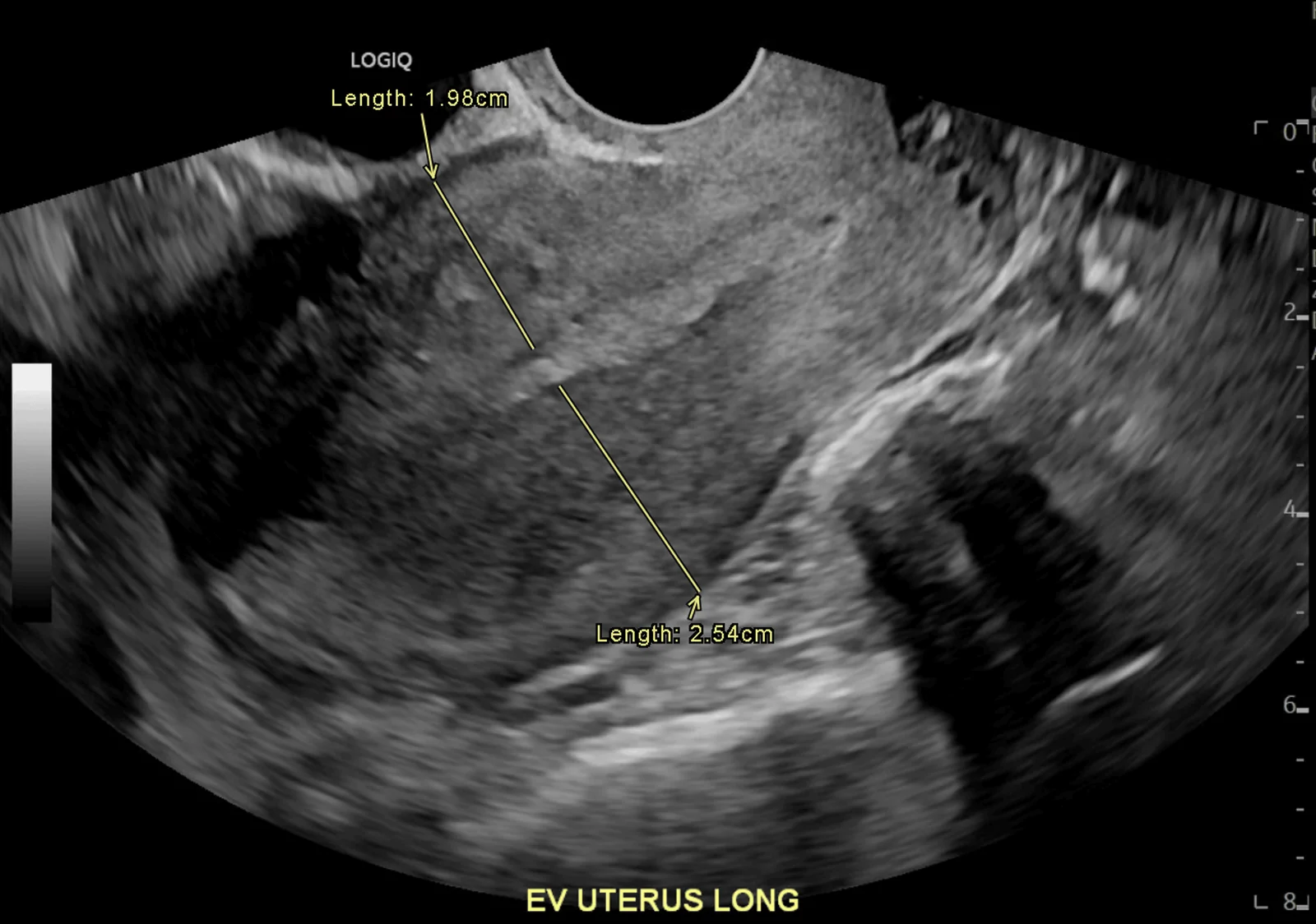
Asymmetric posterior uterine wall thickening with heterogeneous myometrium, which may represent adenomyosis.

**Figure 2:**
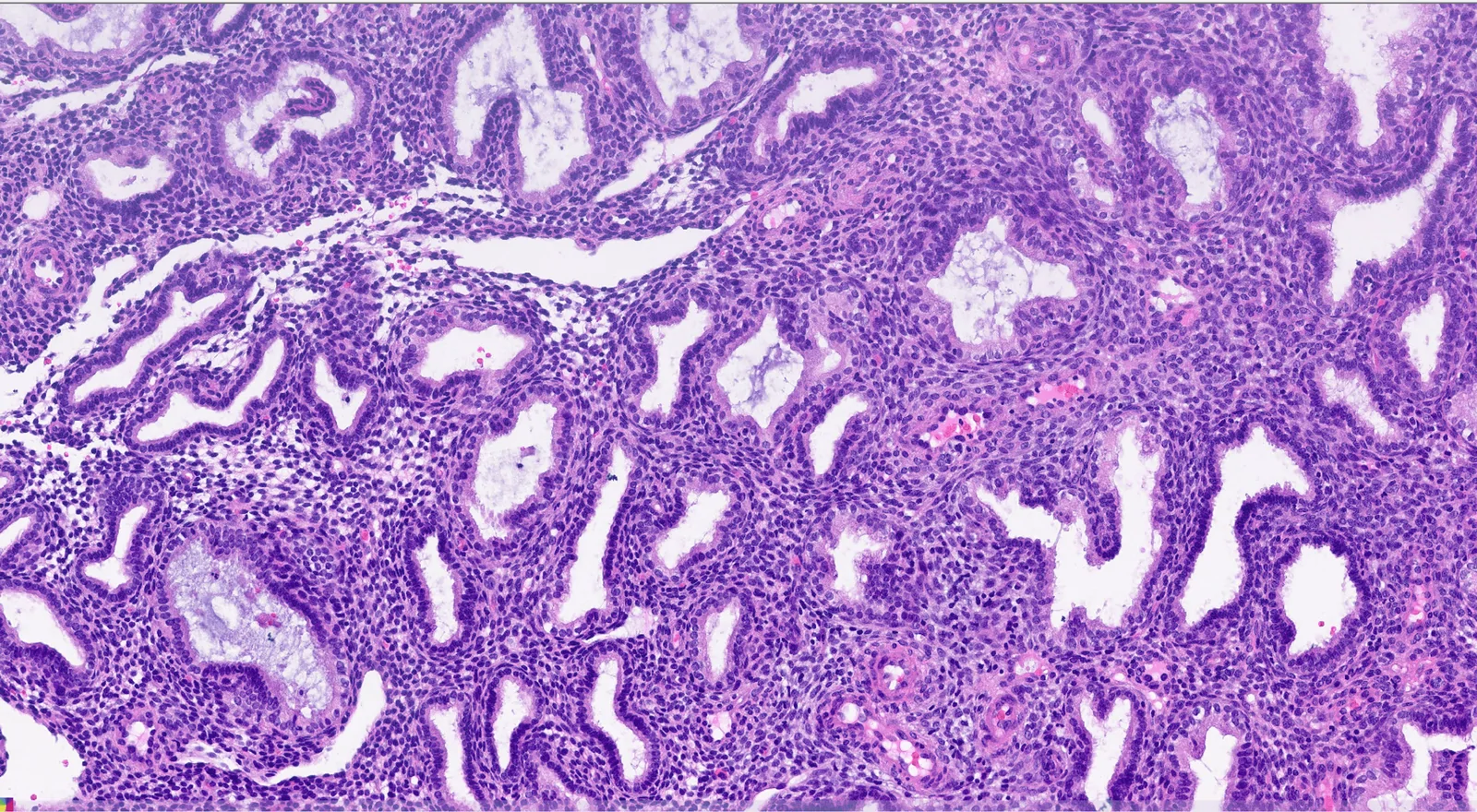
Hematoxylin and eosin (H&E) section demonstrating secretory endometrium with intraluminal secretions, stromal cells with hyperchromatic nuclei, and a high nuclear-to-cytoplasmic ratio.

The patient reported progressively worsening menorrhagia over the preceding two to three years. Menses occurred monthly but were characterized by heavy bleeding lasting one to two weeks, passage of large clots, and severe dysmenorrhea. She reported saturating multiple overnight pads simultaneously and passing golf ball-sized clots. Intermenstrual bleeding was also present. Her obstetric history was gravida 7 para 4-0-3-4 with four prior normal vaginal deliveries. Her bleeding had become severe enough to cause symptomatic anemia requiring blood transfusion during hospitalization. Two weeks before presentation, she began norethindrone therapy; however, bleeding worsened despite medical treatment. Pelvic ultrasound demonstrated findings concerning uterine adenomyosis (Figure 1). An endometrial biopsy performed approximately one week before presentation showed benign findings (Figure 2). Due to persistent AUB refractory to medical management, a hysterectomy was performed.

Gross examination

**Figure 3:**
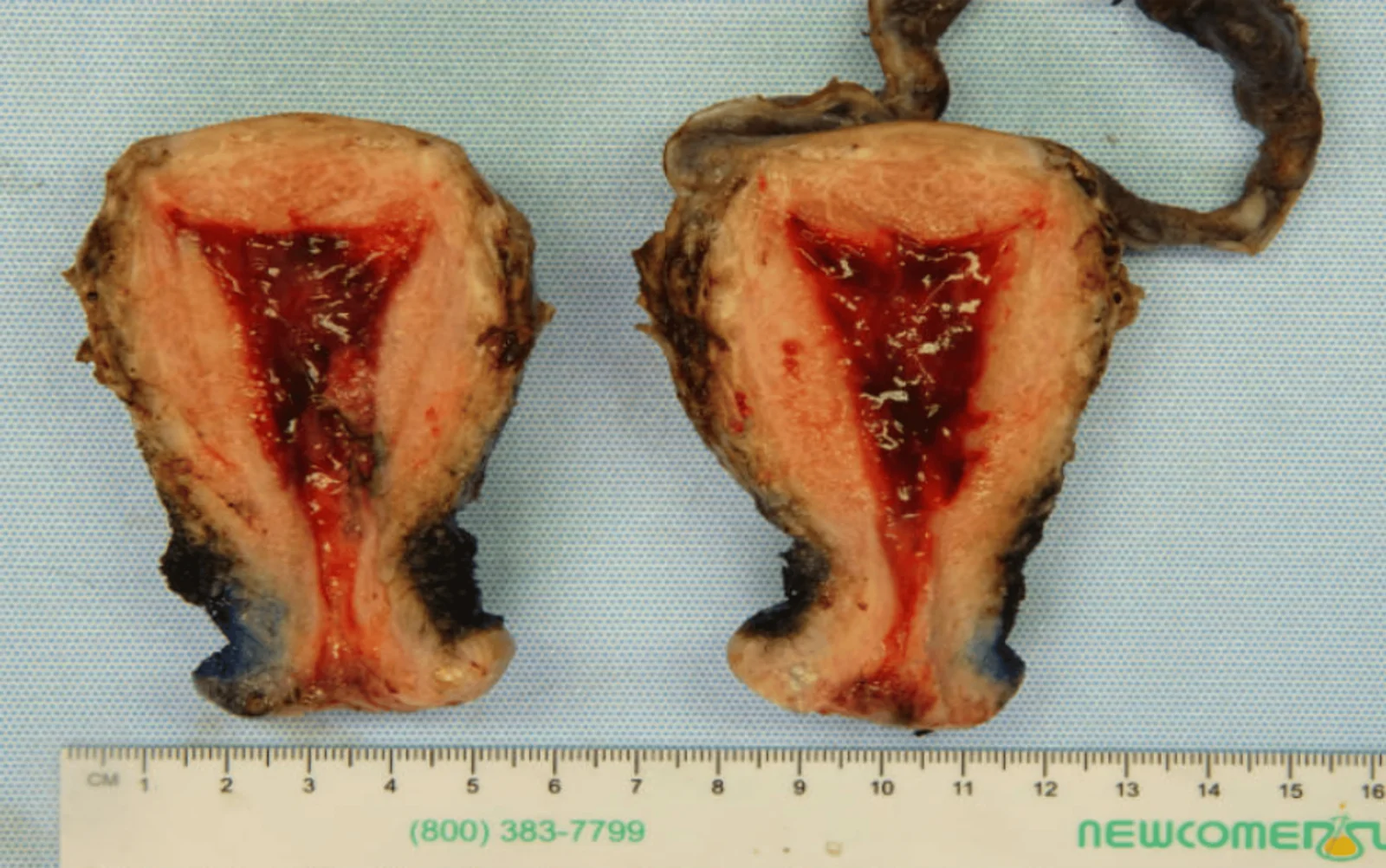
Gross image of a bivalved uterus showcasing a triangular endometrial cavity lined with a purple-red endometrium.

Macroscopic examination of the specimen (Figure 3) showed a uterus measuring 7.3 cm from fundus to ectocervix with attached cervix and bilateral fallopian tubes. The endometrial cavity measured 4.5 cm in length and was lined by purple-red endometrium ranging from 0.1 to 0.4 cm in thickness.

The hysterectomy specimen demonstrated atypical polypoid adenomyofibroma of the endometrium arising in a background of secretory endometrium, with additional findings of adenomyosis and leiomyomata. Examination of the cervix revealed a high-grade squamous intraepithelial lesion (HSIL) involving endocervical glands, with negative surgical margins. High-risk human papillomavirus (HPV) testing demonstrated negative results for HPV types 16 and 18 but positivity for other high-risk HPV subtypes. Given the presence of HSIL and persistent high-risk HPV infection, the patient was placed on long-term surveillance with annual vaginal cytology for 25 years to monitor for potential recurrence of HPV-associated dysplasia in the vaginal epithelium following hysterectomy.

**Figure 4:**
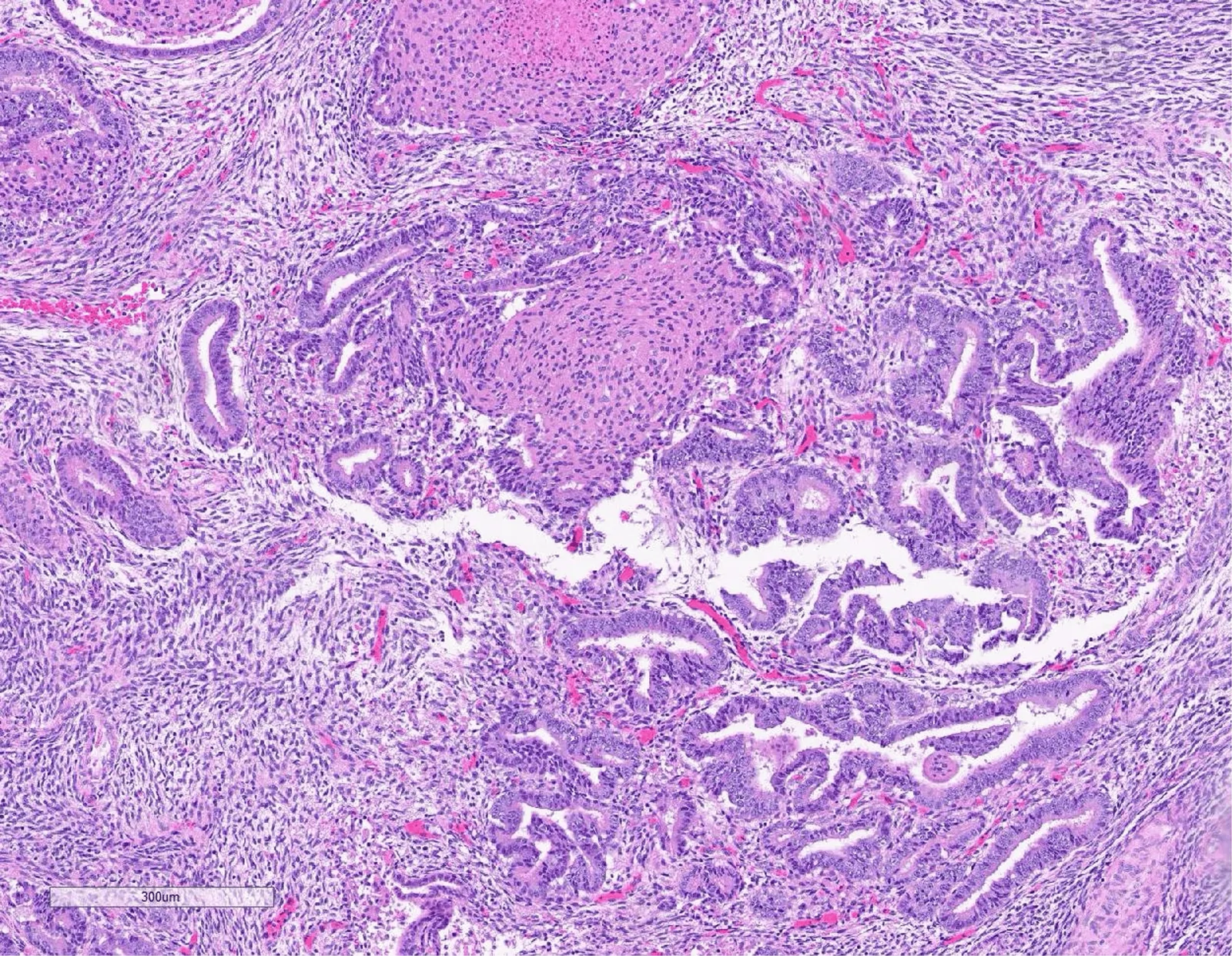
H&E section illustrating atypical endometrial glands with squamous morular metaplasia within a fibromyomatous stroma.

**Figure 5:**
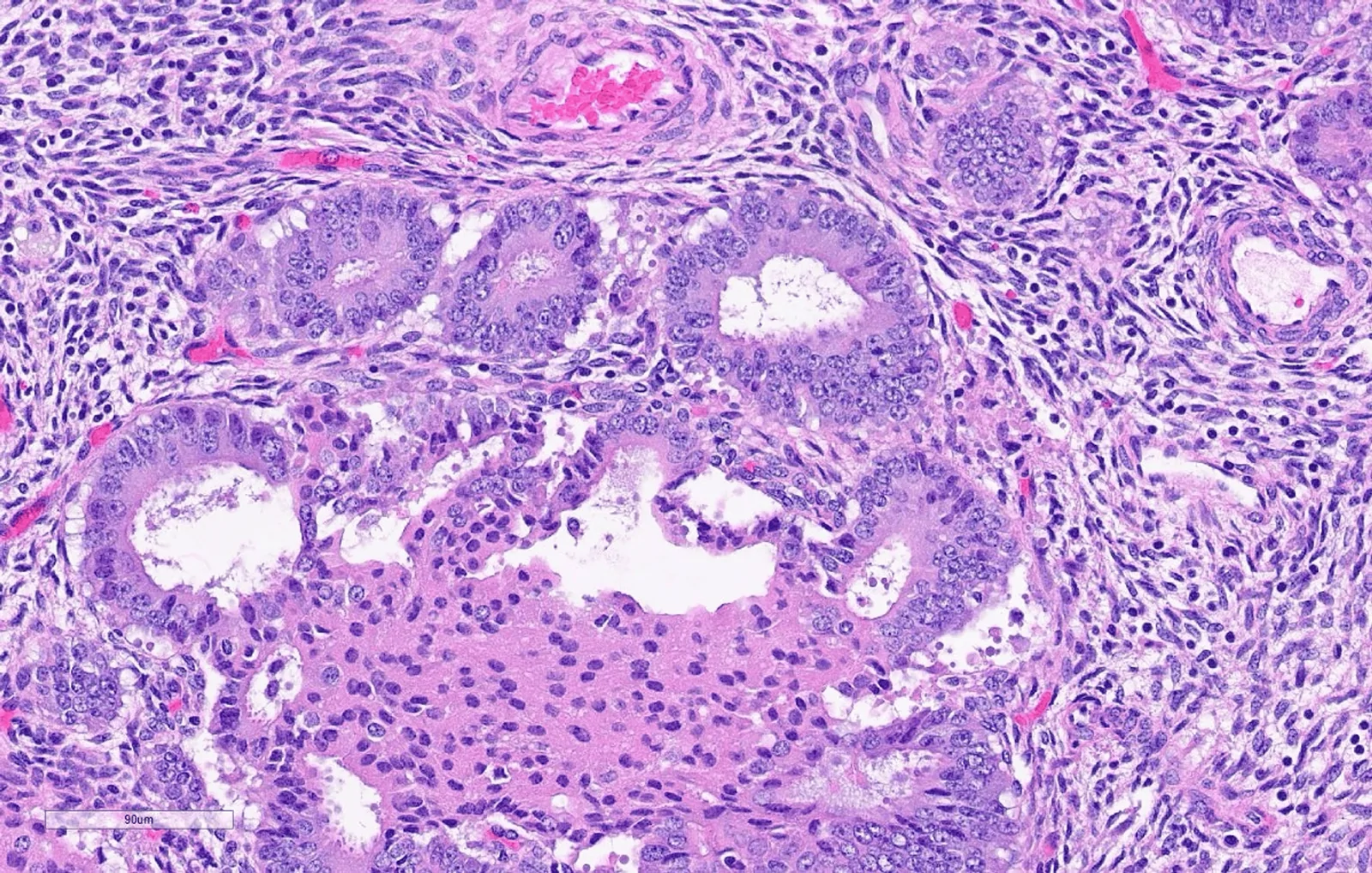
H&E slide revealing florid endometrial glands with architectural complexity, cytologic atypia, and absent mitotic activity.

Histologic examination demonstrated a florid proliferation of atypical endometrial glands embedded within benign fibromyomatous stroma (Figure 4). The glands displayed mild to moderate cytologic atypia (Figure 5), extensive squamous-morular metaplasia, and focal central necrosis. Mitotic activity was minimal. No evidence of glandular crowding, endometrial hyperplasia, or invasive carcinoma was identified.

**Figure 6:**
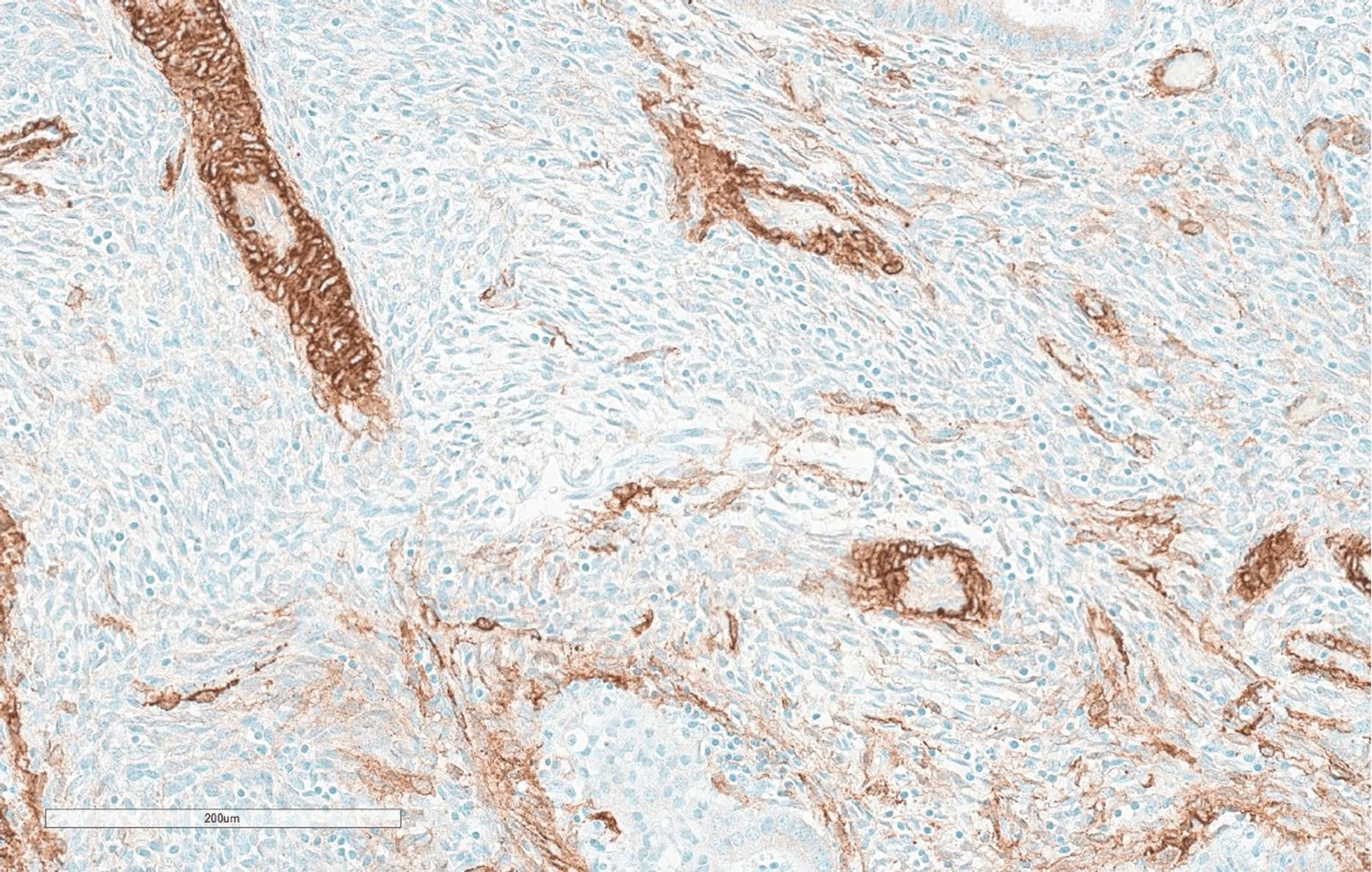
Immunohistochemically, smooth muscle actin is staining positive in the benign myomatous stromal cells.

**Figure 7:**
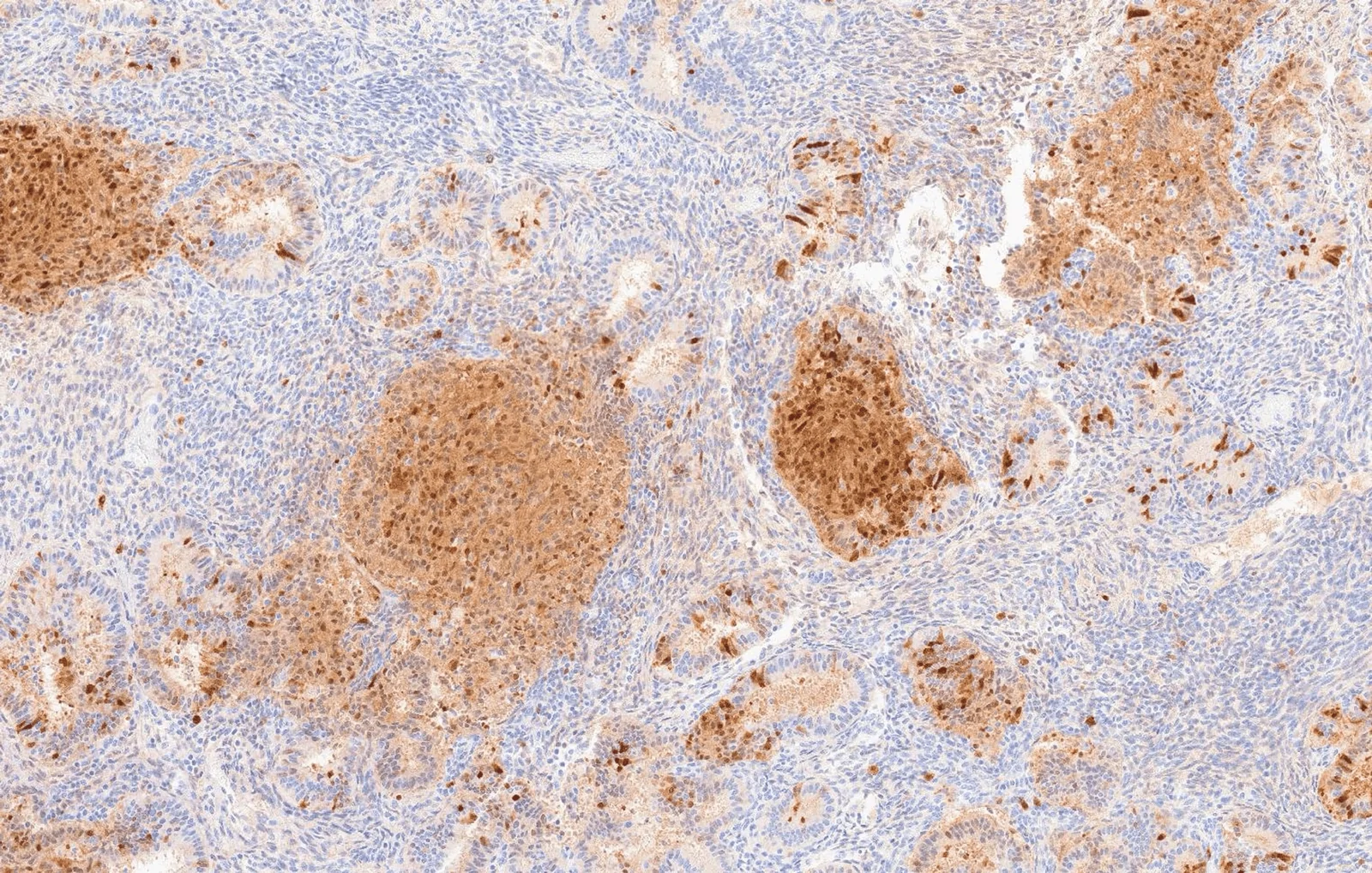
Immunohistochemically, p16 showing diffuse positive staining in the squamous morules and negative staining in the stroma and glands.

**Figure 8:**
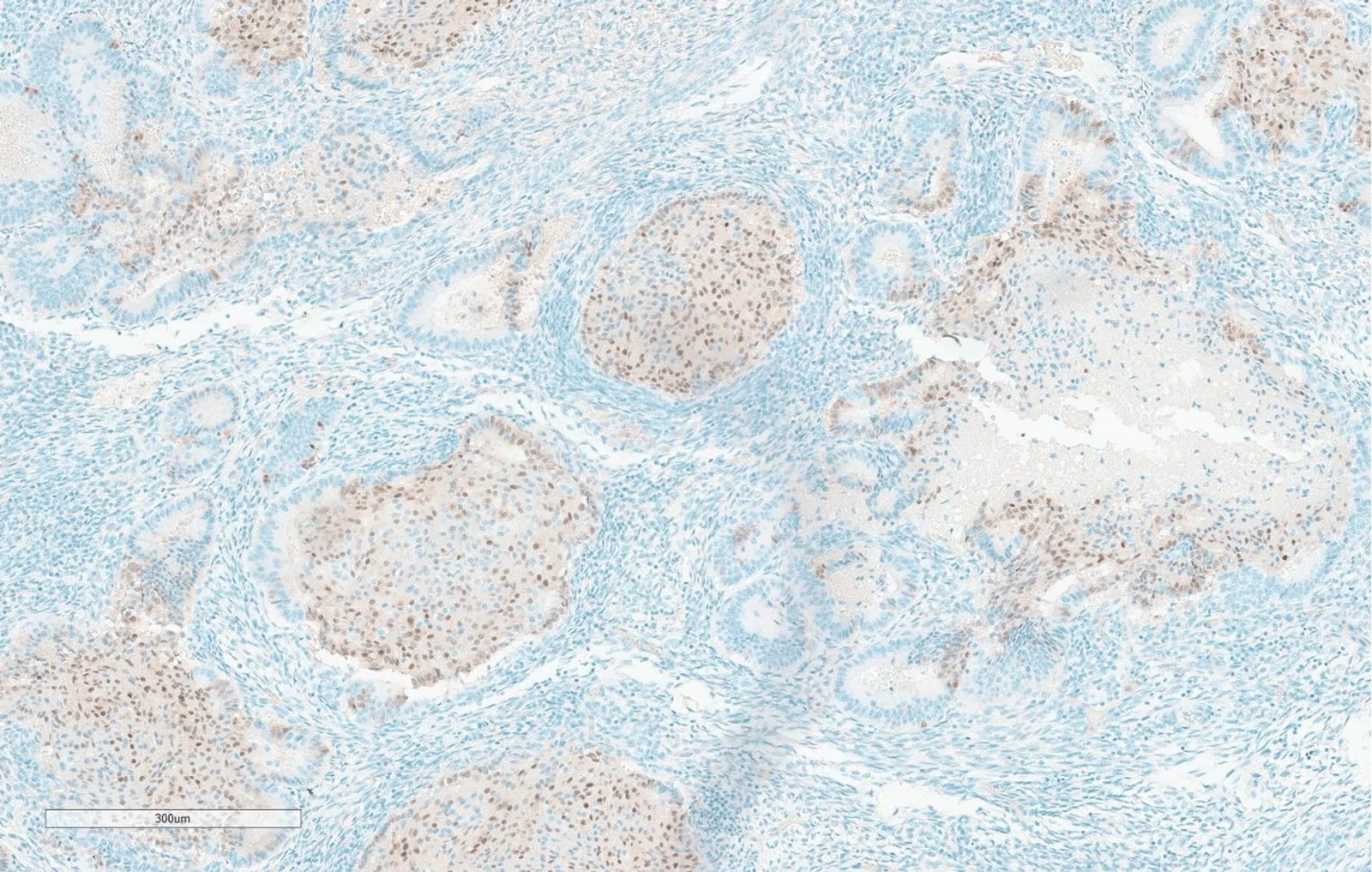
Immunohistochemically, CDX2 showed diffuse positive membranous staining in the squamous morules and focally positive in the glands, but negative staining in the fibromyomatous stromal cells.

**Figure 9:**
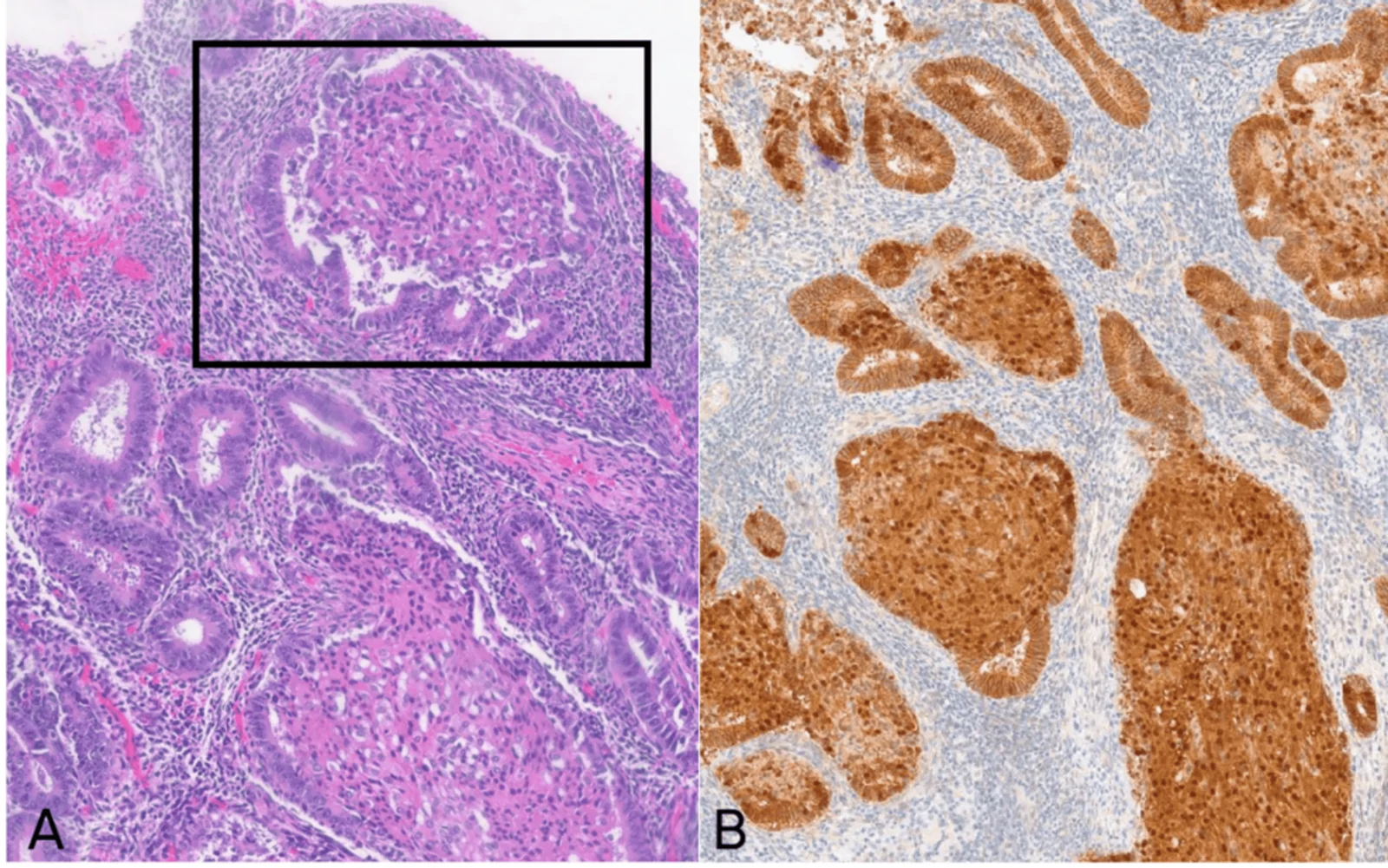
Histopathologic and immunohistochemical findings in atypical polypoid adenomyofibroma. A. H&E-stained section demonstrating atypical endometrial glands with extensive squamous morular metaplasia within a fibromyomatous stroma (squamous morular metaplasia inside the black rectangle). B. β-catenin immunohistochemistry demonstrating strong nuclear staining in the squamous morules and focal nuclear staining in the adjacent atypical endometrial glands.

**Figure 10:**
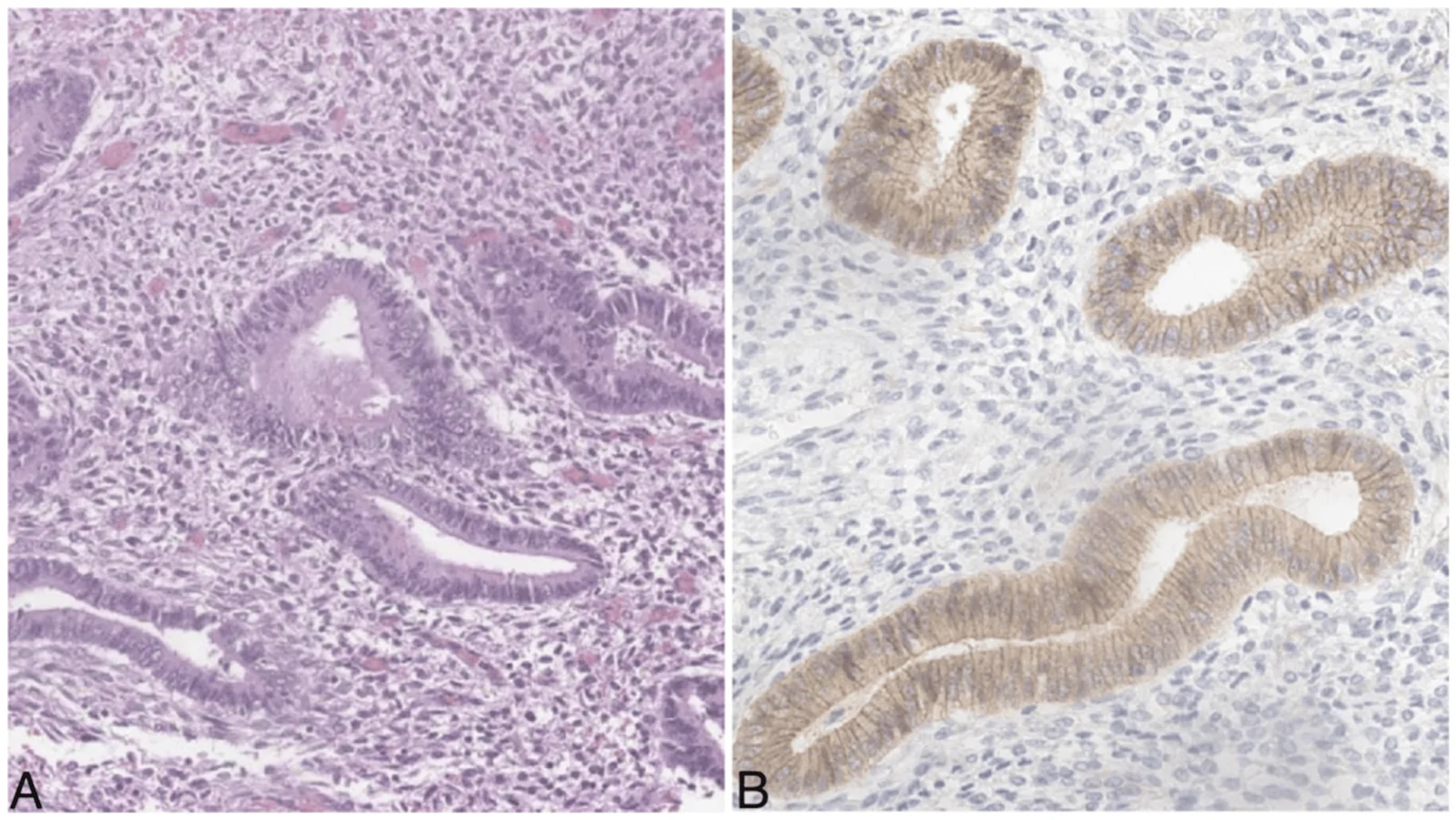
Histopathologic and immunohistochemical findings in background endometrial glands. A: H&E-stained section showing background endometrial glands. B: β-catenin immunohistochemistry displaying a membranous staining pattern among the endometrial glands.

Immunohistochemical analysis revealed focal positivity of the stromal component for smooth muscle actin (SMA) (Figure 6), weak positivity for SATB2, and negativity for desmin. Squamous morules demonstrated positivity for p16 (Figure 7), positivity for CDX2 (Figure 8), and nuclear β-catenin positivity (Figure 9). Membranous β-catenin staining was present within endometrial glands (Figure 10).

Additional findings included adenomyosis, leiomyomata, and HSIL of the cervix involving endocervical glands with negative surgical margins. Bilateral fallopian tubes were unremarkable.

## Discussion

APA is a rare lesion within the spectrum of biphasic endometrial tumors composed of glandular and stromal components. APA shares morphologic overlap with several benign and malignant endometrial conditions, including endometrial polyp (EP), endometrial intraepithelial neoplasia, adenosarcoma, and low-grade endometrioid carcinoma [4,8]. Accurate recognition is therefore important to avoid diagnostic confusion and inappropriate management. APA typically occurs in reproductive-aged females presenting with AUB; most patients are nulliparous, making fertility-sparing management a frequent goal of treatment. A systematic review identified approximately 237 reported cases of APA, underscoring the rarity of these lesions [8]. Fibroma-predominant variants have been reported only sporadically in the literature, and their clinical behavior remains incompletely characterized [3,5,7].

APA must be distinguished from two main mimics: EP and myoinvasive endometrial carcinoma. EP is a common benign polypoid lesion with cystically dilated glands, fibrous stroma, and thick-walled vessels, occurring across a wide age range and presenting from asymptomatic to uterine bleeding. Diagnosis requires at least two of the following three features: altered stroma, irregularly shaped and spaced glands, and thick-walled blood vessels. The stroma is fibrous, and smooth muscle metaplasia is rare, only seen in the stalk. This contrasts with APA, where the stroma is myomatous or fibromyomatous, and squamous metaplasia is prevalent.

Myoinvasive endometrial carcinoma has a mean age of presentation in the sixth decade of life and has a known precursor entity, endometrioid intraepithelial neoplasia. The most common site is the uterine corpus, and the lesion is known to be associated with an increase in estrogen. It presents with AUB and pelvic pain. Microscopically, the glands are closely packed and back-to-back with minimal stroma. Cytology shows nuclear enlargement, loss of polarity, and nuclear rounding. Myometrial invasion is defined by neoplastic glands extending past the endomyometrial junction. Unlike APA, the muscular stroma in myoinvasive endometrial carcinoma is negative for SATB2.

Histologically, the lesions in this case demonstrate atypical endometrial glands embedded within fibromuscular stroma. Squamous morules, as seen here, are a characteristic feature frequently associated with activation of the Wnt/β-catenin signaling pathway [9,10]. Nuclear β-catenin staining within morules is a useful diagnostic clue [9]. The combination of a discrete polypoid lesion composed of atypical glands and squamous morular metaplasia within a benign fibromyomatous stroma supports the final diagnosis of APA. The staining patterns of nuclear β-catenin, smooth muscle actin, and CDX2 further support the diagnosis.

Clinically, AUB is the most common presenting symptom [8]. In this case, the patient experienced progressively worsening menorrhagia that led to transfusion-requiring anemia. Coexisting adenomyosis and leiomyoma likely contributed to the symptom severity and may have masked the underlying endometrial lesion on imaging.

A concurrent HSIL involving the cervix reflected persistent high-risk HPV infection. Although hysterectomy removes the cervix, patients with prior HSIL remain at risk for HPV-related dysplasia in the vaginal epithelium [11]. HPV testing here was negative for types 16 and 18 but positive for other high-risk HPV subtypes. Current cervical cancer screening guidelines recommend continued surveillance for at least 25 years following treatment of HSIL, even in patients who have undergone hysterectomy [11]. Accordingly, this patient was placed on annual vaginal surveillance.

Since APA predominantly affects premenopausal females, fertility-sparing treatments should be offered with close monitoring for recurrent disease, endometrial hyperplasia, and endometrial carcinoma. This is a clinically meaningful concern, as pooled data report overall recurrence and malignant progression rates of 28.9% and 16.6%, respectively [8]. Operative hysteroscopy has a lower relapse rate and risk of cancer development in comparison to uterine dilation, curettage, and polypectomy, with outcomes further improved by combining hysteroscopic resection with adjunctive hormonal therapy [6,8]. Given the lack of standardized management guidelines and common relapse after conservative treatment, patients pursuing fertility preservation require close surveillance with serial hysteroscopy and endometrial biopsy. Postmenopausal patients should be offered a hysterectomy as primary therapeutic treatment.

## Conclusions

APA is a rare biphasic endometrial lesion that can cause severe AUB masquerading as more common etiologies like adenomyosis or leiomyomata, and it may only be diagnosed after hysterectomy. This case highlights the need to consider rare biphasic lesions in unexplained AUB and the importance of comprehensive histologic evaluation to distinguish APA from premalignant and malignant mimics, avoiding both overtreatment and undertreatment. The coexisting cervical HSIL also underscores the need for long-term surveillance in patients with persistent high-risk HPV infection.
